# Navigating neonatal thrombosis: contemporary insights into a complex disorder in a unique population

**DOI:** 10.3389/fphar.2026.1900164

**Published:** 2026-09-29

**Authors:** Rozeta Sokou, Vasiliki Mougiou, Alexandra Lianou, Andreas G. Tsantes, Stefanos Bonovas, Sotirios P. Fortis, Effie G. Papageorgiou, Anastasios G. Kriebardis, Argirios E. Tsantes, Nicoletta Iacovidou

**Affiliations:** 1 Neonatal Department, National and Kapodistrian University of Athens, Aretaieio Hospital, Athens, Greece; 2 Neonatal Unit, Royal Victoria Infirmary, Newcastle upon Tyne, United Kingdom; 3 Neonatal Intensive Care Unit, “Aghios Panteleimon” General Hospital of Nikea, Piraeus, Greece; 4 Laboratory of Haematology and Blood Bank Unit, “Attikon” Hospital, School of Medicine, National and Kapodistrian University of Athens, Athens, Greece; 5 Department of Biomedical Sciences, Humanitas University, Milan, Italy; 6 IRCCS Humanitas Research Hospital, Milan, Italy; 7 Laboratory of Reliability and Quality Control in Laboratory Hematology (HemQcR), Department of Bio-Medical Sciences, School of Health & Caring Sciences, University of West Attica (UniWA), Athens, Greece

**Keywords:** developmental hemostasis, neonatal thrombotic disorders, neonates, metabolic health, thromboembolic disease, thrombosis risk

## Abstract

Neonatal thromboembolic disease (TE) is an increasingly recognized clinical condition associated with significant morbidity, despite its relatively low incidence in the general paediatric population. Neonates-especially those in Neonatal Intensive Care Units (NICUs)-are at the highest risk for TE among all paediatric age groups. This elevated risk is attributed to the developmental characteristics of neonatal haemostasis, the frequent use of central venous catheters, and the presence of significant underlying comorbidities. Over recent decades, the reported incidence of neonatal TE has risen significantly, reflecting improvement in neonatal intensive care, and increased awareness and recognition of thrombotic complications. The unique pathophysiology of neonatal TE, and its distinct risk factors, underscore the need for a stronger neonatal-specific evidence to support prevention, diagnosis and management of thromboembolic events. The objective of this review is to summarise and critically appraise existing evidence on the epidemiology, the pathophysiology and the risk factors related to neonatal TE. It also aims to identify current knowledge gaps and areas requiring further investigation. Overall, the available evidence supports a multifactorial model of neonatal thrombosis, whereby developmental hemostasis interacts with acquired antenatal and perinatal risk factors. However, substantial heterogeneity in epidemiological data and diagnostic approaches continues to hinder accurate thrombotic risk stratification and evidence-based clinical decision-making.

## 1 Introduction

Thromboembolic disease (TE) in childhood occurs relatively rarely compared to adulthood, with an estimated incidence of venous thromboembolism (VTE) ranging from 0.07 to 0.14 per 10,000 children (Andrew et al., 1994; Van Ommen et al., 2001; Robinson et al., 2021). In this review, TE is used as an overarching term for arterial and venous thrombotic events occurring during the neonatal period, whereas “thrombosis” is used when referring to thrombus formation or to specific thrombotic manifestations. Within the paediatric population, neonates form the group that is most likely to present either with an isolated, asymptomatic thrombotic event or with severe TE. In this context, it is important to distinguish between thrombotic events, which encompass all instances of pathological clot formation regardless of embolization, and TE, a clinical syndrome in which a thrombus forms within the vasculature and subsequently embolizes a distant vessel, resulting in occlusion and tissue ischemia (Sokou et al., 2026a).

Severely or even critically ill neonates-term and preterm-are at increased risk of developing symptomatic TE. During the first month of life, the risk of thrombotic complications, including thrombotic events or severe TE, is approximately 40 times higher than during any other period of childhood, especially in critically ill neonates or in those with central venous catheters (CVCs) (Viviana Bacciedoni MDa et al., 2016; Greenway et al., 2004; Schmidt and Andrew, 1995). Compared with other paediatric subpopulations, critically ill neonates, including preterm infants, face a disproportionately high risk of TE complications due to the coexistence of multiple prothrombotic risk factors. These include the developmental immaturity of their haemostasis, the frequent need for invasive medical procedures, and the presence of multiple underlying comorbidities (Monagle et al., 2006).

The incidence of TE in this particularly vulnerable population-especially those admitted to NICUs-has an increasing trend (Robinson et al., 2021; Guzman et al., 2023; Raffini et al., 2009). Between 1997 and 2018, it was reported that the incidence of neonatal TE among all liveborn infants presented an almost thirteen-fold increase, with an increase of more than six-fold among those hospitalized in NICUs (Raffini et al., 2009; Nowak-Göttl et al., 1997; Saracco et al., 2016a; Betensky et al., 2022; Song et al., 2023a). This rise has been attributed to several factors like the improved survival rates of critically ill and/or preterm neonates, the more frequent use of CVCs, and an overall increased awareness of TE and its associated risk factors in this population (Robinson et al., 2021; Raffini et al., 2009; Saracco et al., 2016a; Setty et al., 2012).

Neonatal thrombotic disorders are generally classified as either inherited or acquired, with the overwhelming majority of the cases (approximately 95%) being caused by acquired factors (Guzman et al., 2023; Paes et al., 2022; Bhat et al., 2018). The main acquired risk factors are the presence of a CVC, sepsis, prolonged hospitalization, and congenital heart disease (Robinson et al., 2021; Paes et al., 2022; Bhat et al., 2018; Abdelsamei et al., 2024; Sirachainan et al., 2018). Inherited risk factors encompass thrombophilic conditions, such as gene mutations of Factor V Leiden (FVL) and prothrombin gene (PTG) G20210A, as well as deficiencies of protein C, protein S, and AT (Guzman et al., 2023; Egami et al., 2021).

TE events in neonates have significant morbidity and mortality, including the loss of vascular access that is vital to them, an increased risk of bleeding related to anticoagulant therapy, and long-term complications (Sokou et al., 2026a). These complications include limb loss secondary to ischemia in cases of arterial thrombosis, and the development of portal hypertension, renal dysfunction, and post-thrombotic syndrome in cases of venous thrombosis (Andrew et al., 1994; Raffini et al., 2009; Setty et al., 2012; Morag et al., 2006; Ouellette et al., 2020).

The pathophysiology and many of the risk factors associated with neonatal TE events are unique to this age group, underlining the need for the development of guidelines for the prevention and management of TE specifically tailored to neonates (Makatsariya et al., 2022). Despite the growing recognition of the incidence and clinical significance of thromboembolic events in neonates, the heterogeneity of thromboembolic events and the variability of their clinical presentations hinder the systematic investigation of risk factors, management strategies, and long-term outcomes (Guzman et al., 2023; Bhat and Monagle, 2020). Current recommendations for the management of neonatal thrombosis are largely derived from extrapolating data from adult and older paediatric populations, small observational studies with limited numbers of neonates, expert consensus, and existing clinical guidelines (Monagle et al., 2025a).

Available data regarding optimal prevention and treatment strategies for this condition in neonates remain limited. Understanding the multifactorial nature of neonatal TE is essential for improving prevention, early diagnosis, and therapeutic management. The complex interplay between congenital factors, such as the unique characteristics of developmental hemostasis, and acquired risk factors, including the use of intravascular catheters, infections, and prematurity, contributes to the heterogeneity and clinical complexity of the condition (Sokou et al., 2026a).

However, the scarcity of robust, high-quality data in the neonatal population limits a comprehensive understanding of the underlying pathogenetic mechanisms and the relative contribution of individual thrombotic risk factors. A critical synthesis of the available data is therefore needed to delineate what is currently established and where important uncertainties remain. Therefore, the present review aims to critically appraise existing evidence on the epidemiology, pathophysiology, and risk factors associated with neonatal TE, while identifying key gaps in current knowledge that restrict the translation of available evidence into clinical practice and may inform future neonatal specific prevention, diagnostic and management strategies.

For this narrative review, a literature search was conducted in the PubMed and Scopus databases in order to identify relevant publications on neonatal thrombosis. The search focused on the literature addressing the epidemiology, pathophysiology, developmental hemostasis, and risk factors associated with thrombotic events in neonates. In addition, the reference lists of relevant articles were screened to identify additional pertinent publications. The search included publications available up to and including April 2026. Consistent with the narrative nature of the review, the literature was selected based on its relevance to the scope of the review, with particular emphasis on large epidemiological studies and multicentre registries, systematic reviews and meta-analyses, clinical practice guidelines and consensus/communication statements from scientific societies, as well as recent studies providing neonatal-specific data.

## 2 Epidemiology

In neonatal and paediatric populations, thrombosis remains largely underdiagnosed (Levy-Mendelovich et al., 2023). The estimated annual incidence of arterial or VTE in neonates and children ranges between 2.6 and 6.4 cases per 100,000 individuals (Betensky et al., 2022; Bhatt and Chan, 2021). It was clearly established that the incidence of VTE in the paediatric population follows a bimodal age distribution pattern, with infants <1 year of age, particularly neonates, being most frequently affected (Andrew et al., 1994; Van Ommen et al., 2001). Neonates are considered to be at particularly high risk, as a great proportion of paediatric TE cases occur in patients admitted to NICUs.

International registries estimated the incidence of thrombosis in neonates to be between 0.29 and 0.51 per 10,000 neonates overall, increasing to 24 per 10,000 in those admitted to NICUs (Van Ommen et al., 2001; Schmidt and Andrew, 1995; Nowak-Göttl et al., 1997). In a large national U.S. database with 1,158,755 NICU admissions, the incidence of neonatal thrombosis was 0.20% (20 per 10,000 admissions) (Robinson et al., 2021). Factors independently associated with thrombosis included prematurity, male sex, congenital heart disease, sepsis, respiratory support, vasopressor therapy, CVC placement, and other invasive procedures. Thrombosis had higher mortality rates (11% vs*.* 2% in neonates without thrombosis) and prolonged length of hospitalization (median 57 vs. 10 days).

A multicentre case–control study analysed data from 31 level IV NICUs in the US (126,894 neonates admitted between 1 January 2010, and 13 December 2016), using the Children’s Hospital Neonatal Database, and reported an incidence of TE events of 15.5/1,000 admissions (95% CI 14.8–16.2), of which 11.5/1,000 were venous and 4.8 per 1,000 were arterial. Of the 1,326 recorded cases, 77.1% were venous and 27.3% were arterial thromboses while 4.4% involved both types (Bhat et al., 2022). A prospective Egyptian cohort study consisting of 886 neonates admitted to NICU reported a TE incidence of 40/1,000 admissions, with portal vein thrombosis (PVT) being the leading subtype (30.6%) (Abdelsamei et al., 2024). Notably, 69% of neonates with thrombosis had genetic mutations, most commonly affecting FVL (52.3%). Significant risk factors included sepsis, the presence of a central venous line, and prolonged NICU hospitalization. The overall mortality in this cohort reached 52.8% (Abdelsamei et al., 2024).

The Canadian Neonatal Network analysed data from 39,971 neonates admitted to NICU between January 2014 and December 2016, and calculated the incidence of neonatal thrombosis to be 15/1,000 admissions (El-Naggar et al., 2020). VTE were most commonly identified in the portal vein (34%), whereas arterial thromboses were primarily located in the cerebral arteries (34%). Management of neonatal thrombosis varied according to the type and anatomical location of the thrombotic event. Most cases of cerebral arterial thrombosis (44/51, 86%), cerebral venous thrombosis (24/39, 61%), and other CVC-associated VTE (311/491, 63%) were treated conservatively. In contrast, low-molecular-weight heparin (LMWH) was the principal therapeutic modality for arterial thromboses located outside the cerebral and renal vasculature (39/93, 42%) and for renal vein thrombosis (RVT) (13/27, 48%). Unfractionated heparin (UFH) was another therapeutic choice employed in 16.6% (25/151) of arterial thromboses and in 8.7% (48/550) of venous thromboses (El-Naggar et al., 2020).

An analysis of the first episodes of systemic TE in 75 neonates (0–28 days), as recorded by the Italian Registry of Paediatric Thrombosis-the largest European registry for neonatal and paediatric thrombosis-from neonatal centres between January 2007 and July 2013, showed that 55% of cases were VTE, 29% arterial, and 16% intracardiac (Saracco et al., 2016b). Most of the affected neonates were preterm (71%) and male (65%). Early onset of thrombosis (<24 h of life) was associated with maternal or placental morbidity in 70% of the cases and with inherited thrombophilia in 33% of the cases. Overall mortality was 15%, highlighting the significance and potential burden of early neonatal thrombosis.

More recent data from the Italian Registry of Paediatric Thrombosis, reported 1,001 cases in neonates and children between 1996 and 2022 (Pelizza et al., 2023). Of these, 57.8% were male and 42.2% female, with a median age of occurrence of thrombosis of 0.9 years. Overall, 24.8% presented with neonatal cerebral thromboembolism, 8.7% with neonatal systemic TE, 41.5% with paediatric cerebral TE, and 25.1% with paediatric systemic TE. The high recorded frequency of cerebral thrombosis demonstrates the importance of early diagnosis. A national Japanese study estimated an incidence of 0.39 per 10,000 liveborn neonates, with intracranial lesions identified in 66 of 77 cases. Protein C variants were associated with an increased risk of disability (odds ratio 5.74, 95% CI 1.16–28.4; P = 0.03), but not with mortality (Egami et al., 2021).

Historically, the majority of TE events in neonates correspond to VTE, including deep vein thrombosis (DVT) and pulmonary embolism (PE) (Guzman et al., 2023). In a prospective Canadian study, 61% of TE cases were VTE, 31% and arterial thrombotic events, while 4% presented with a combination of venous and arterial thrombosis (Schmidt and Andrew, 1995). Bhat et al., analysed data from the Children’s Hospital Neonatal Database and demonstrated that 77% of cases were VTE and 27% were arterial thrombotic events (Bhat et al., 2022). Analysis of data collected and extracted from a clinical data library, which prospectively captures and integrates data from daily medical notes, nurses’ documentation, and other clinician-generated entries through a computer-assisted documentation system, showed that the incidence of paediatric VTE increased exponentially over the past 30 years (1997–2015), and the greatest rise was observed in infants, particularly those admitted to NICUs (Robinson et al., 2021).

A retrospective cohort study based on the Paediatric Health Information System (PHIS) database included patients from birth to <18 years of age hospitalized between 2008 and 2019 and had an ICD-9-CM or ICD-10-CM diagnosis code for VTE (O'Brien et al., 2022). They reported that the rate of VTE in hospitalized paediatric patients continues to rise, given that there was a 130% increase observed between 2008–2019, vs*.* a 70% increase that had been previously noted between 2001–2007. The observed increase most likely reflects enhanced awareness of the condition, improvement in the care of critically ill children, and the extended use of lifesaving albeit invasive technologies, such as CVCs, in neonates (O'Brien et al., 2022).

Detailed data from the most recent studies reporting the incidence of neonatal thrombosis, as presented in Table 1, reveal significant heterogeneity in study design, study populations, risk factors, and clinical outcomes. Diagnostic criteria and methodologies vary considerably in those studies, and differences in detecting and reporting cases substantially influenced the reported incidence rates. Studies differ in their use of clinical, laboratory, and imaging assessments with some implementing systematic imaging surveillance, such as Doppler ultrasonography or computed tomography/magnetic resonance (CT/MR) angiography, in all high-risk neonates, while others rely on symptom-driven evaluation. This variability contributes to the underdiagnosis of asymptomatic cases (Paes et al., 2022; Abdelsamei et al., 2024; Sirachainan et al., 2018; Bhatia et al., 2022).

**TABLE 1:** Summary of studies reporting the incidence of neonatal thrombosi**s**.

Study	Country	Type/Size of study	NICU admissions	Incidence	Case ascertainment	Type of thrombosis	Primary site	Important risk factors	Mortality
Robinson et al. (2021)	United States of America	Retrospective multicentre cohort (Pediatrix Medical Group NICUs)	1.158.755	0.20% (20/10.000)	Clinician-documented thrombosis; no systematic imaging surveillance reported	Venous/Arterial	All	Prematurity, male gender, congenital heart defects, sepsis, mechanical ventilation, inotropic administration, central venous catheters, invasive procedures	11% vs. 2%
Bhat et al. (2022)	United States of America	Multicentre (31 centres)	126.894	15,5/1000	ICD-9 code-based identification of diagnosed thrombosis	Venous 11,5/Arterial 4,8	–	–	–
Bhatia et al. (2022)	Canada	Retrospective single-centre chart review	4.860	38/1000	Database identification plus individual chart and imaging confirmation	–	–	Central venous catheters, sepsis, RDS	–
Paes et al. (2022)	–	Retrospective single-centre cohort study	234 neonates with thrombosis	25/1000	Diagnosed thrombosis identified through retrospective medical-record review; imaging used for diagnosis and follow-up	–	Portal Vein 59,4%	Central venous catheters 73,1%, preterm/term 1,5:1	–
Abdelsamei et al. (2024)	Egypt	Prospective two-centre cohort study	886	40/1000	Clinically suspected thrombosis confirmed by site-specific imaging	–	Portal Vein 30,6%	Genetic mutations 69% (Factor V Leiden 52,3%), sepsis, central venous catheters, prolonged hospital stay	52,8%
El-Naggar et al. (2020)	Canada	Retrospective multicentre cohort (Canadian Neonatal Medicine Network)	39.971	15/1000	Clinically diagnosed thrombosis recorded in the Canadian Neonatal Network database	Venous/Arterial	Venous: Portal vein, Arterial: Encephalic arteries	–	–
Saracco et al. (2016b)	Italy	Multicentre registry (Italian Registry of Thrombosis in Infants, RITI)	75 neonatal TE cases; incidence denominator available for 6,254 NICU admissions from 2 centres	5.8 and 6.6/1,000 NICU admissions	Symptomatic or asymptomatic thromboembolism confirmed by imaging	Venous 55%, Arterial 29%, Endocardiac 16%	–	Early onset <1day, maternal/placental disease 70%, inherited thrombophilia 33%	15%
Sirachainan et al. (2018)	Thailand	Retrospective single-centre study	2.463	120 per 10,000	ICD-10 code-based identification supplemented by Hematology Unit consultation records	– Arterial ischemic stroke 36%; arterial thrombosis 29%; deep vein thrombosis 14%; cerebral venous sinus thrombosis 11%; purpura fulminans 7%; renal vein thrombosis 3%	-	Central catheter 35.7%; congenital heart disease 17.9%; infection 14.3%; maternal factors 10.7%; protein C deficiency	14,3%
Egami et al. (2021)	Japan	Retrospective nationwide survey	−744 NICU facilities surveyed; 120 reported cases; 77 cases included in detailed analysis	3.91/100,000 live births	Physician-diagnosed thromboembolism with imaging confirmation; nationwide questionnaire-based surveillance	Intracranial thrombohemorrhage 85.7%; purpura fulminans 6.5%; deep vein thrombosis 6.5%; ocular bleeding 3.9%	Intracranial lesions 66/77(85.7%)	Central venous catheter 32.5%; maternal disease 20.8%; placental/umbilical cord abnormalities 13.0%; family history of thromboembolism 6.5%	–

Also, the incorporation of genetic testing for thrombophilia varies across studies, as in some cases it is performed only in selected patients, thus affecting the detection of inherited risk factors (Saracco et al., 2016a; Abdelsamei et al., 2024). Population selection and the intensity of surveillance are major sources of heterogeneity. Large studies based on national registries often rely on administrative data and ICD coding, which can lead to underreporting or erroneous classification of cases, while single-centre studies with active surveillance report higher incidence rates due to more frequent imaging and heightened clinical awareness (Robinson et al., 2021; Paes et al., 2022; Abdelsamei et al., 2024; Sirachainan et al., 2018; El-Naggar et al., 2020; Bhatia et al., 2022). Furthermore, the definition of thrombosis itself (e.g., inclusion of catheter-related events, arterial, venous, or intracardiac thromboses), as well as the minimum duration of NICU hospitalization required for study inclusion, differ between study designs and further affect incidence estimates (Robinson et al., 2021; Bhat et al., 2022).

A systematic comparison of these criteria and methods indicates that studies employing more rigorous and standardized diagnostic protocols, incorporating systematic imaging surveillance and a more complete assessment of risk factors, consistently report higher incidence rates compared with studies relying on passive or administrative data collection (Paes et al., 2022; Bhat et al., 2018; Abdelsamei et al., 2024; Sirachainan et al., 2018; Saracco et al., 2016b; Bhatia et al., 2022). Therefore, differences in detection and reporting methodologies constitute a pivotal factor that determines the observed variability in the published incidence rates of neonatal thrombosis.

## 3 Pathophysiology and risk factors

As early as 1845, Rudolf Virchow described three fundamental factors contributing to the development of thrombosis-known today as Virchow’s triad-which include (Figure 1): (1) disorder of blood flow (stasis), (2) vascular injury, and (3) alterations in blood composition (hypercoagulability) (Virchow, 1989). This theory remains, to some extent, applicable to the interpretation of the aetiology of thrombotic events in both adults and children. The increased predisposition to TE complications observed in preterm and critically ill neonates can be explained by the coexistence of the factors in Virchow’s triad (endothelial injury, blood stasis, and hypercoagulability), in combination with the distinct characteristics of the neonatal haemostatic system (Kenet et al., 2018).

**FIGURE 1:**
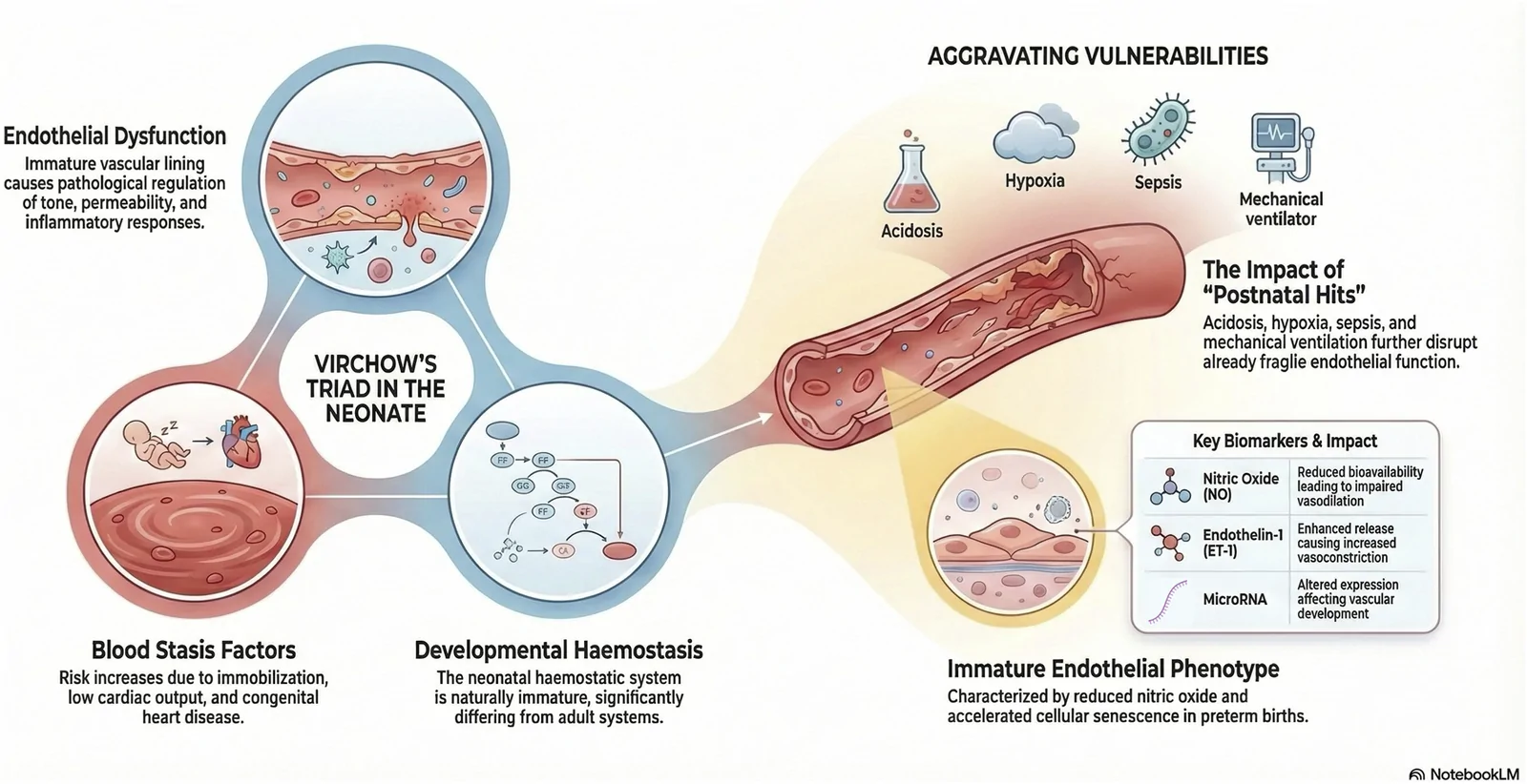
Virchow’s Triad in Neonatal Thrombosis. An overview of how blood flow disorder (stasis), vascular injury (endothelial dysfunction), and hypercoagulability converge in preterm and critically ill neonates. Immature developmental hemostasis, combined with prenatal and postnatal clinical stressors, significantly elevates their risk for thromboembolic events. Created with NotebookLM.

Immobilization, low cardiac output, congenital heart disease, alterations in blood viscosity, and the hemodynamic instability that characterizes ill and preterm neonates lead to decreased physiological blood flow within the vascular lumen, thereby increasing the risk of stasis and activation of the coagulation cascade (Bhat and Monagle, 2012). Endothelial dysfunction in neonates consists of disturbances in the structural and functional properties of the vascular endothelium during the neonatal period, resulting in pathological regulation of the vascular tone, the vascular permeability, the inflammatory responses, and the haemostatic balance (Bhat and Monagle, 2012). In neonates, especially those born preterm or exposed to adverse intrauterine conditions (such as maternal preeclampsia), endothelial cells display an immature endothelial phenotype, which is characterized by:

- reduced nitric oxide (NO) bioavailability,
- increased vasoconstriction due to enhanced release of endothelin-1 (ET-1),
- alterations in microRNA expression
- and accelerated cellular senescence

Furthermore, postnatal aggravating factors (“postnatal hits”), such as acidosis, hypoxia, hyperglycaemia, mechanical respiratory support, and sepsis, further disrupt endothelial function, thus increasing the risk of thrombosis (Andrew et al., 1987). One of the key pathogenetic mechanisms of neonatal thrombosis is the immaturity of the neonatal haemostatic system, which differs significantly from that of older children and adults. In healthy neonates, thrombosis is generally rare; however, due to developmental haemostasis, preterm neonates, particularly those who are critically ill, are at increased risk of thromboembolic complications (Andrew et al., 1987) (Figure 1).

### 3.1 Developmental haemostasis

The term developmental haemostasis refers to the gradual process of maturation of the haemostatic system, from foetal life through adulthood (Andrew et al., 1987). Coagulation factors do not cross the placenta but are synthesized *in utero* from early stages of foetal development. Foetal haemostasis begins at approximately 5 weeks of gestation, with the hepatic synthesis of procoagulant factors (e.g., factors VII, VIII, IX, and X), anticoagulant proteins (protein C and antithrombin [AT]) (Monagle et al., 2025a), and fibrinogen, which are detectable in plasma as early as the 10th-20th week of gestation (Jaffray and Young, 2013; Sokou et al., 2023a; Sokou et al., 2024a).

Compared with adults, neonates demonstrate both quantitative and qualitative differences in multiple components of the haemostatic system. Although most of these differences are balanced out within the first 6 months of life, some persist to adolescence (Sokou et al., 2023a; Sokou et al., 2025a). In term neonates, the plasma concentrations of several procoagulant proteins, particularly vitamin K-dependent factors and contact system factors, are reduced compared to adult levels.

More specifically, the activities of factors II, VII, IX, and X, as well as the concentrations of factors XI and XII, reach approximately 50% of adult levels at birth and rise up to adult levels by 6 months of age (Sokou et al., 2023a). At birth, factor V and fibrinogen levels are within the normal adult reference range, whereas that of factor VIII (FVIII) are increased (Ignjatovic et al., 2011). Accordingly, anticoagulant factors levels - AT, heparin cofactor II, and proteins C and S -are also reduced at birth (Sokou et al., 2023a; Aarts et al., 1983; Jones et al., 1985). Most of these anticoagulant proteins reach adult levels by 3 months of age, except for protein C, which remains at lower levels until 6 months of age. In contrast, α2-macroglobulin, the main thrombin inhibitor in neonates, is present at birth at levels nearly twice as high as these observed in adults and remains increased until approximately 6 months of age (Sokou et al., 2023a).

Differences are also observed in the fibrinolytic system, which is involved in clot breakdown. Levels of plasminogen and tissue plasminogen activator (tPA) are lower in neonates, whereas plasminogen activator inhibitor (PAI) levels are increased, resulting in a hypofibrinolytic state (Sokou et al., 2023a; Suarez et al., 1984). In addition, platelet function is reduced; however, primary haemostasis may be enhanced due to elevated levels of von Willebrand factor (VWF) and a greater proportion (compared to adults) of functional, high–molecular-weight VWF multimers, leading to increased platelet adhesion (Balasundaram, 2025; Sola-Visner, 2012).

Newborns have a complex haemostatic defect strongly related to gestational age, vitamin K stores, the maturity of hepatic function, and the developmental stage of the vascular endothelium (Sokou et al., 2023a; Suarez et al., 1984). Importantly, several of the differences that characterize the neonatal haemostatic system counterbalance one another, thus maintaining a dynamic equilibrium between coagulation and fibrinolysis. Therefore, the physiologically lower levels of natural anticoagulants should not, in isolation, be interpreted as an inherently prothrombotic state. As a result, the neonatal haemostatic system remains balanced and functional under physiological conditions, although with limited reserves when this equilibrium is challenged by acquired clinical triggers such as perinatal hypoxia, inflammation, or sepsis, particularly in ill and preterm neonates (Sokou et al., 2023a; Suarez et al., 1984).

Neonates exhibit a specific risk factor profile for the development of thrombosis, due to the contribution of maternal and perinatal factors that are not encountered in other paediatric age groups. More than half of neonates with a TE event have one or more maternally derived risk factors for thrombosis (Saracco et al., 2016b). Overall, the factors contributing to the occurrence of TE complications in neonates can be classified into 3 categories: maternal/placental, perinatal, and neonatal factors (Table 2).

**TABLE 2:** Categories and subgroups of risk factors for the development of neonatal thrombosis.

Risk factor category	Specific risk factors	References
Prenatal (Maternal/Placental)	Maternal infection Placental conditions (i.e. abruption, placental insufficiency) Gestational Diabetes Melitus or pre-existing Hypertension, Preeclampsia, Dyslipidaemia Metabolic syndrome Antiphospholipid syndrome Congenital thrombophilia Premature rupture of membranes	(Guzman et al., 2023; Saracco et al., 2016a; Saracco et al., 2016b; O'Brien et al., 2022; Easterlin et al., 2022; Amankwah et al., 2014)
Perinatal Factors	Perinatal asphyxia Trauma during labour Prematurity Low Birth weight	(Van Ommen et al., 2001; Saracco et al., 2016a; O'Brien et al., 2022; Easterlin et al., 2022; Amankwah et al., 2014)
Post-natal factors (neonatal)	Central venous catheter Sepsis Inflammatory conditions (i.e. necrotising enterocolitis) Dehydration Congenital heart defects Hepatic dysfunction Thrombophilia (inherited or acquired) Need for mechanical ventilation Haemodynamic instability Transfusion of full blood or other blood products Surgery	(Van Ommen et al., 2001; Saracco et al., 2016a; O'Brien et al., 2022; Sokou et al., 2023a; Easterlin et al., 2022; Amankwah et al., 2014)

### 3.2 Maternal factors and foetal thrombotic angiopathy

Maternal factors such as infection, placental disorders, diabetes mellitus, hypertension, preeclampsia, dyslipidaemia, metabolic syndrome, antiphospholipid syndrome, inherited thrombophilia, and premature rupture of membranes are implicated and associated with foetal thrombotic vasculopathy (FTV) (Kraus, 2013). FTV is a thrombotic vascular disorder characterised by thrombosis of arteries and veins in the foeto-placental circulation, leading to ischemic changes in villi distal to the site of occlusion and destruction of the villous vascular layer (Aarts et al., 1983; Kraus, 2013; Pathak et al., 2011; Sokou et al., 2025b). FTV, if extensive, may lead to a reduction in the functional reserve of the placenta. or may be associated with additional thrombotic or TE events in the systemic circulation of the foetus itself (Kraus, 2013).

In severe cases, the former may result in intrauterine growth restriction (IUGR) or intrauterine foetal demise, whereas the latter can cause neonatal TE, neurological injury, and systemic thrombosis in the neonate, with infarctions affecting various foetal structures, such as the brain, kidneys, or, more rarely, an extremity (e.g., an arm or a leg) (McDonald et al., 2004; Redline, 2005; Magnetti et al., 2014; Heider, 2017; Chisholm and Heerema-McKenney, 2015) (Figure 2). FTV cannot be detected prenatally, and diagnosis is established exclusively through histopathological examination of the placenta postnatally.

**FIGURE 2:**
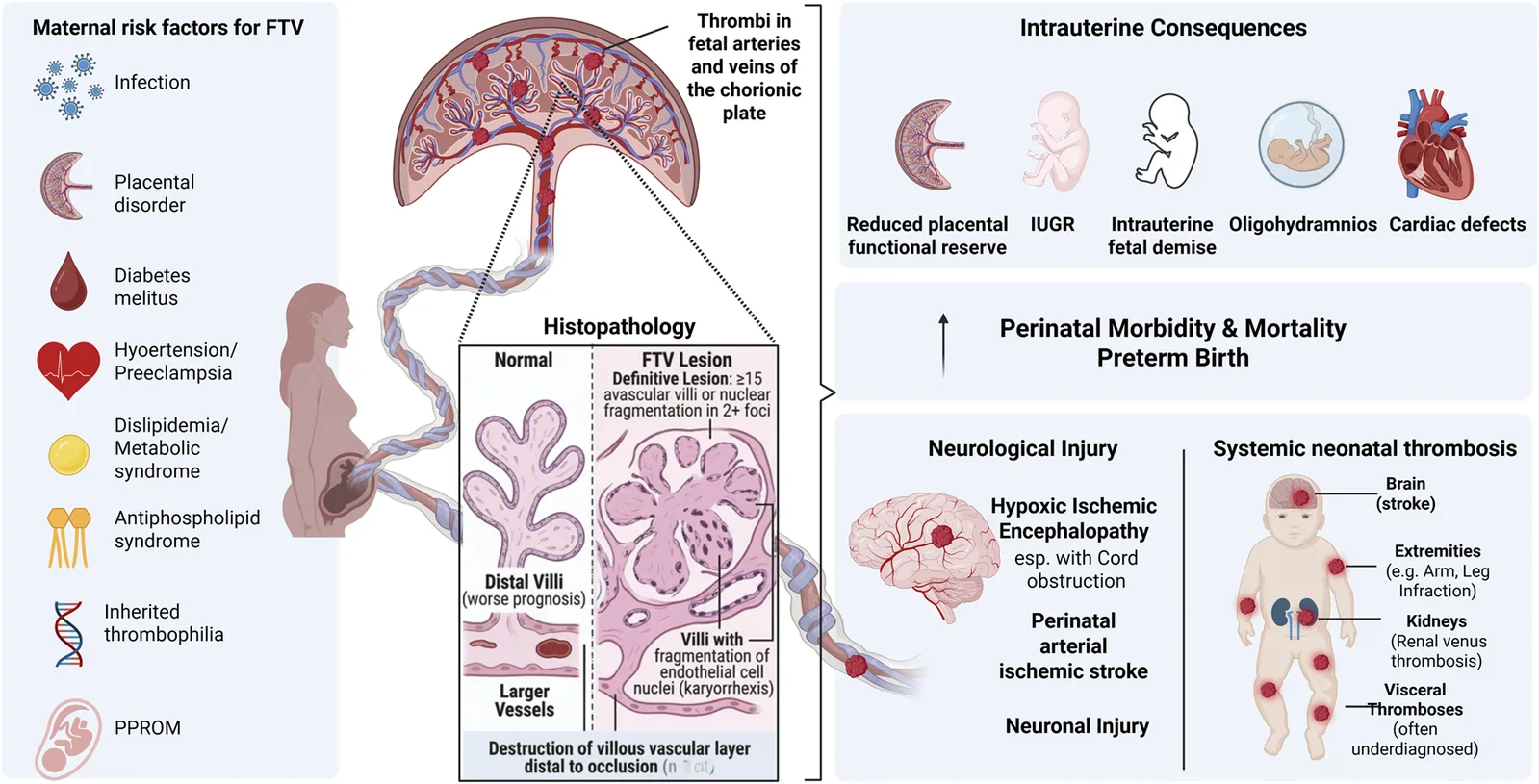
Pathogenesis and clinical outcomes of Fetal Thrombotic Vasculopathy (FTV). This diagram illustrates the pathogenesis of FTV, including the maternal risk factors, the histopathological changes within the foeto-placental circulation, and the resulting clinical consequences, including intrauterine growth restriction (IUGR), neurological injury, and systemic neonatal thrombosis. PPROM: preterm premature rapture of membranes. Created in BioRender. L., A. (2026) https://BioRender.com/p50pymu.

In 2004, the Foetal Vascular Obstruction Nosology Committee formulated a concise and standardized definition of FTV. According to this definition, FTV is characterized by the presence of 15 or more avascular villi, or villi showing fragmentation of the nuclei of endothelial and other nucleated cells of the foetal circulation in two or more foci per histologic section, with or without detectable lesions in the foetal vessels, and in the absence of “villitis of unknown aetiology with inflammation of stem villi and avascular villi” (Redline et al., 2004).

The presence of extensive lesions (≥2 foci with ≥15 avascular villi or destruction of the villous vascular stroma per histologic section) is associated with an increased risk of IUGR, neurological injury, neonatal thrombosis, and increased perinatal morbidity and mortality (Chisholm and Heerema-McKenney, 2015). These lesions are clinically more significant if located in distal villi, as they carry a worse prognosis for the neonate compared with lesions affecting the larger placental vessels (Stanek, 2022). It is described that placentas affected by FTV are associated with a ninefold higher incidence of intrauterine foetal demise, a sixfold higher incidence of cardiac defects, an almost sixfold higher incidence of oligohydramnios, and a twofold higher incidence of IUGR compared with normal placentas (Saleemuddin et al., 2010). In addition, the risk for preterm birth is also increased (Saleemuddin et al., 2010).

FTV is also an important cause of hypoxic–ischemic encephalopathy (HIE)-, particularly if associated with obstructive issues of the umbilical cord (Wintermark et al., 2010a). A study of autopsies from intrauterine foetal deaths that compared neuropathology with placental pathology reported that FTV is associated with neuronal injury (nuclear fragmentation) in the brain (Chang et al., 2011). In a study including 186 neonatal stroke cases from the Canadian Paediatric Ischemic Stroke Registry, placenta was examined in only 12 patients, out of which 6 had thrombi in the umbilical cord, the vessels of the chorionic plate, or other characteristics consistent with FTV (Elbers et al., 2011). Placental examination from neonates with perinatal arterial ischemic stroke (PAIS) suggests that thrombotic and inflammatory lesions, reduced placental function, and acute destructive damage to placental structure might contribute to the pathogenesis of neonatal thrombotic events (Kraus, 2013).

FTV constitutes a significant risk factor for neonatal stroke or other TE events, particularly when multiple predisposing factors coexist in the mother, the foetus, or the perinatal environment. It is a rare but clinically important vascular disorder of the foetal circulation, with a prevalence ranging from 0.3% to 6.4% (Redline et al., 2004; Redline and Pappin, 1995; Leistra-Leistra et al., 2004; Lepais et al., 2014). FTV is associated with an increased risk of neonatal thrombosis, including renal venous thromboses and of other organs, which are not always clinically evident after birth and therefore remain undiagnosed, thus impairing the growth and function of the affected organs (Kraus, 2013).

It was proposed that placentas from neonates who develop thromboembolic events during the first days of life should always undergo examination, given their strong association with FTV (Makatsariya et al., 2022; Magnetti et al., 2014). Overall, FTV highlights the importance of careful placental examination, especially in neonates with intrauterine complications or neonatal thrombosis, as the identification of lesions can provide critical information for diagnostic evaluation, understanding of the mechanisms underlying thrombosis, and prevention of future complications. Future studies with larger, well-controlled cohorts are indispensable to define the contribution of FTV to the pathogenesis of neonatal stroke and other thrombotic complications with higher precision.

The presence of inherited thrombophilias, such as AT deficiency (ATD), protein C deficiency (PCD), protein S deficiency (PSD), and mutations in the FVL gene and the PTG G20210A mutation, or acquired thrombophilic disorders in the mother are associated with an increased risk of placental thrombotic lesions, including FTV (Kupferminc, 2005; Ferriero et al., 2019; Rambaldi et al., 2014; Tuckuviene et al., 2012). Acquired thrombophilic disorders are important predisposing factors for neonatal thrombosis and include antiphospholipid syndrome (characterized by the presence of antiphospholipid antibodies-APA, such as lupus anticoagulant, anticardiolipin antibodies, and anti–β2-glycoprotein I antibodies) and homocysteinemia due to disorders of homocysteine metabolism. The presence of these acquired disorders in the mother increases the risk of placental thrombotic vasculopathy and consequently that of neonatal thrombosis (Kupferminc, 2005; Ferriero et al., 2019; Rambaldi et al., 2014).

Maternal thrombophilia contributes to an increased thrombotic tendency during pregnancy *via* multiple mechanisms; pregnancy itself induces a hypercoagulable state, with increased levels of procoagulant factors (e.g., factor VIII, fibrinogen, VWF), reduced levels of natural anticoagulants (e.g., protein S), and decreased fibrinolytic activity due to elevated levels of PAI (Warren et al., 2023). In the presence of maternal thrombophilia, these changes are amplified, leading to a prothrombotic state that promotes microvascular thrombosis within the placental circulation (Kupferminc, 2005; Warren et al., 2023). This results in the development of placental vasculopathy, including FTV, which reduces blood flow to the foetus and increases the risk of neonatal thrombosis, particularly in the renal and other visceral veins (Makatsariya et al., 2022; Gogia and Machin, 2008).

The association between FTV and maternal or foetal thrombophilias is not as strong as initially proposed by case reports and early uncontrolled studies. More recent controlled prospective studies showed that the proportion of maternal thrombophilias in pregnancies complicated by FTV is similar to that in pregnancies without FTV, indicating that the presence of thrombophilia itself does not significantly increase the incidence of FTV (Rodger et al., 2014; Simcox et al., 2015; Stanek, 2021; Bates et al., 2012). Regardless of the extent to which thrombophilia directly contributes to the development of FTV, previously studied haemostatic disorders appear to function primarily as predisposing factors. Clinically significant placental thrombosis typically arises from the interaction of these predisposing factors with secondary, usually prenatal events, such as inflammatory processes, intrauterine hypoxia, or mechanical disturbances of blood flow.

When present, genetic or acquired thrombophilic factors increase placental susceptibility to such insults, thereby amplifying the likelihood of activation of local thrombotic mechanisms and the development of clinically significant FTV (Redline, 2004). The presence of thrombophilias most likely acts synergistically with other, stronger predisposing factors for thrombosis, such as reduced foetal perfusion due to umbilical cord obstruction, foetal circulatory stasis (e.g., heart failure, hypercoagulability), or placental tissue injury, that leads to activation of the endothelium, platelets, and the coagulation system through inflammatory mechanisms (Redline, 2004; Wintermark et al., 2010b; Gomez et al., 1998; Matzinger, 2002).

### 3.3 Neonatal factors

The most common neonatal factors are associated with the presence of CVC and the presence of sepsis or other inflammatory conditions (Easterlin et al., 2022; Amankwah et al., 2014).

#### 3.3.1 Neonatal thrombophilia

The role of neonatal thrombophilia in the development of neonatal TE remains under-defined. Thrombophilia in neonates, as in adults, may be either inherited or acquired. In neonates and infants with idiopathic VTE, and in paediatric populations in whom thrombosis is associated with underlying medical conditions, inherited thrombophilia (IT) is identified as a significant predisposing factor (Guzman et al., 2023; Makatsariya et al., 2022; Nowak-Göttl et al., 2011). The most common congenital thrombophilias associated with increased thrombotic risk include ATD, PCD, PSD, as well as the FVL and PTG G20210A mutations (Makatsariya et al., 2022; Limperger et al., 2016).

Activated protein C (APC) is a vitamin K–dependent anticoagulant protein that inactivates the activated forms of factors V (FVa) and VIII (FVIIIa) (Dahlbäck, 1995a). The FVL mutation results in resistance to activated protein C, thereby creating a prothrombotic state (Dahlbäck, 1995b). The presence of the FVL mutation is associated with an approximately 5- to 7- fold increased risk of thromboembolism in heterozygous adults and an approximately 80 times higher risk in homozygote adults (Rosendaal et al., 1995; Zöller et al., 1997; Kang et al., 2025). Although the FVL mutation predisposes to VTE, the clinical utility of routine screening remains controversial. While most carriers of the FVL mutation experience their first thromboembolic event at an older age, meta-analyses demonstrated a statistically significant association between the FVL mutation and neonatal cerebral sinus venous thrombosis (CSVT) (Laugesaar et al., 2010).

The PTG mutation constitutes the second most common type of inherited thrombophilia, with a prevalence of approximately 1%–2% in the White European population (Poort et al., 1996). The mutation leads to increased prothrombin levels and is associated with a twofold to threefold increased risk of thromboembolism in adults (Junker et al., 1999). Although sporadic cases of neonatal thrombosis associated with this mutation were reported, current evidence does not show a strong association between the PTG G20210A mutation and neonatal thromboembolic disease (Laugesaar et al., 2010).

AT is a serine protease inhibitor that inactivates coagulation factors IIa (thrombin), IXa, Xa, XIa, and XIIa (Lane and Caso, 1989; Kállai et al., 2024). ATD may be acquired, as in congenital nephrotic syndrome, or inherited. Heterozygous ATD was reported in cases of neonatal arterial and venous thrombosis without being typically associated with purpura fulminans. Instead, it was associated with thrombotic events in unusual locations, such as the coronary arteries, the aorta, and the central nervous system (Jochmans et al., 1994; Peeters et al., 1993).

Homozygous ATD is very rare but has been implicated in severe venous and arterial thromboses in neonates. Cases that were reported include neonatal thrombosis of the pulmonary artery, inferior vena cava, abdominal veins, and peripheral limb veins (Swoboda et al., 2017; Rodgers and Mahajerin, 2023). The therapeutic management includes anticoagulant therapy, however, heparin may be ineffective, as its anticoagulant action depends on the presence of AT (Chaireti et al., 2020). AT concentrate is a safe and effective measure for the acute management of hereditary ATD with thrombosis in neonates; nevertheless, routine prophylactic use in preterm infants or for acquired ATD is not supported and may be associated with adverse outcomes.

Recent data on the safety and efficacy of AT concentrate use in neonates with ATD indicate that AT replacement is effective in enhancing AT activity and facilitating anticoagulant therapy, especially in neonates with hereditary deficiency and active thrombosis or in those requiring treatment with UFH (Monagle et al., 2025a; Alami et al., 2024). In a large paediatric cohort that included neonates, administration of AT concentrate resulted in increased AT levels and improved anti–factor Xa activity, and no bleeding, thrombotic, or allergic events were reported within 1 week of administration, supporting a favourable short-term safety profile (Balasundaram, 2025). Data regarding long-term safety remain limited, and its use should be restricted to clearly defined indications, with close monitoring of efficacy and potential adverse events (Rodgers and Mahajerin, 2023).

Lipoprotein (a) [Lp(a)] consists of a polypeptide chain linked to a low-density lipoprotein (LDL) particle and demonstrates structural homology with plasminogen. Because of this similarity, Lp(a) competes with plasminogen in its binding to fibrin, resulting in reduced fibrinolytic activity. Elevated Lp(a) levels can significantly limit fibrinolysis, thereby increasing the risk of thrombosis. Although paediatric venous and arterial throm (El-Naggar et al., 2020) botic events were reported, the precise risk of neonatal thrombosis associated with elevated Lp(a) has not been fully established. Nevertheless, it seems that increased Lp(a) levels contribute to the occurrence of neonatal ischemic arterial stroke (PAIS) (Günther et al., 2000; Nowak-Göttl et al., 1999).

Data from follow-up on VTE recurrence in neonates indicate recurrence rates of approximately 3% in provoked or secondary cases, and up to 21% in idiopathic VTE. Beyond underlying medical conditions, recent studies in paediatric VTE showcased significant associations with Factor V G1691A and PTG G20210A mutations, and with deficiencies of protein C, protein S, and AT. This association is even more pronounced in cases with combined inherited thrombophilias, underlining the role of genetic predisposition in VTE recurrence in neonates (Nowak-Göttl et al., 2011). Regardless of age at first VTE episode, the ORs for individual inherited thrombophilic defects ranged from 2.4 for the PTG G20210A mutation to 9.4 in neonates and infants with ATD. The overall OR for the persistent presence of APA was 4.9 in paediatric patients with venous VTE. Furthermore, the PTG G20210A mutation (OR: 2.1), as well as deficiencies of protein C (OR: 2.4), protein S (OR: 3.1), and AT (OR: 3.0), are significant predisposing factors for VTE recurrence in the paediatric population (Nowak-Göttl et al., 2011).

Published data suggest an association between inherited thrombophilia and the development of thrombosis in the paediatric population (Nowak-Göttl et al., 2011). In a meta-analysis, the presence of thrombophilia was significantly associated with an increased risk of VTE in neonates and children, however, approximately 70% of affected patients had at least one additional prothrombotic clinical factor, indicating that thrombophilia *per se* may not be sufficient for the development of thromboembolism in neonates (Andrew et al., 1987).

In an Israeli–German prospective cohort study that included paediatric patients at high risk for VTE-carriers of ATD, PCD, and PSD-over a 10-year period of follow-up, the absolute risk of recurrence per 100 person-years was 5.4% in paediatric carriers of ATD, 1.3% in children with PCD, 0.7% in those with PSD, and 0.9% in children without thrombophilia (Limperger et al., 2016). In the same study, the mortality rate during recurrence was 1.3%, and children with ATD, PCD, and PSD at the time of the first VTE episode or arterial ischemic stroke (AIS) did not differ in terms of age, sex, or the proportion of provoked versus unprovoked thromboembolic events. Patients with a positive family history of VTE were fewer in the group without thrombophilia at the time of the first thrombotic episode, however, family history did not influence the risk of recurrence. The recurrence rate was significantly higher in paediatric carriers of ATD compared to individuals with PCD, PSD, or no thrombophilia. ATD and female sex were highlighted as independent prognostic factors for recurrence, with hazard ratios (HRs) of 6.5 and 2.6, respectively (Limperger et al., 2016).

In studies such as that by Aschka et al. (1996), the frequency of the FVL mutation in 125 neonates and children with VTE episodes was increased in the age groups 0–6 months (26%) and 10–18 years (30%). The presence of the FVL mutation was identified in 6 of 22 neonates with arterial thrombosis of the central nervous system in the study by Hagstrom et al. (1998), while data from Zenz et al. (1998) detected no FVL mutations among 11 neonates with ischemic stroke, whereas the mutation was present in 6 of 22 children with stroke beyond the neonatal period, suggesting an age-dependent association with pediatric ischemic strokeas a risk factor for ischemic stroke in children of Austrian origin beyond the neonatal period.

On the contrary, the PTG G20210A mutation did not constitute an independent risk factor for stroke in the same population. Saracco and colleagues reported that up to 33% of infants with “early-onset” VTE, defined as VTE occurring on the first day after birth, had an inherited thrombophilia (Saracco et al., 2016b). Revel-Vilk et al. (2003), in a cohort study of children with VTE, did not demonstrate a difference in the overall prevalence of inherited thrombophilia between neonates with VTE and the healthy paediatric population, suggesting that routine screening for inherited thrombophilia in neonates with VTE may not be necessary when a clear inducing factor is present.

It is also worth mentioning some extremely rare but particularly severe forms of thrombotic microangiopathy in neonates, with the most characteristic example being thrombotic thrombocytopenic purpura (TTP) which is caused by pathogenic mutations in the *ADAMTS13* gene that lead to a significant reduction or complete absence of its protein activity (Hellström-Westas et al., 2005). Deficiency of the ADAMTS13 enzyme in plasma leads to the accumulation of ultra-large multimers of VWF, which causes thrombotic microangiopathy and thrombocytopenia. TTP may occur either as congenital ADAMTS13 deficiency, or as a result of developing antibodies against the enzyme that block its activity (Strauss et al., 2017).

Regarding VWF levels in neonates, most studies report that they are increased after birth. Factors like stress and inflammation further stimulate VWF expression, which partially explains its transient increase during the neonatal period (Sokou et al., 2023a). However, it was found that an isolated reduction in ADAMTS13 levels or an isolated increase in VWF are not sufficient to cause TE complications (Papadogeorgou et al., 2023). Thrombosis usually develops in the presence of additional risk factors, such as hypoxia, sepsis, acute illness, or the presence of a CVC (Papadogeorgou et al., 2023). In any case, a disruption of the normal relationship between VWF and ADAMTS13 levels is considered an important independent pathogenetic factor for the development of neonatal thrombosis (Papadogeorgou et al., 2023).

To date, the role of testing for inherited thrombophilia in children remains a matter of debate. Although risk factors that are related with morbidity in neonates represent the most common cause of TE events, in cases of isolated VTE in a neonate without identifiable risk factors, recurrent VTE, or a positive family history it is recommended to use a stepwise approach to investigate the presence of inherited thrombophilia (Sokou et al., 2026a). Thrombophilia testing in parents may be useful for the interpretation of findings, while maternal testing for APA possibly has a diagnostic value (Mot et al., 2009). The pathogenesis of perinatal thrombosis associated with APA can be explained both by transplacental transfer of maternal antibodies and by *de novo* antibody production by the neonate (Gordon et al., 2014; Tzatzadaki-Amantou et al., 2026). Although the presence of maternal APA is not an independent risk factor for neonatal thrombosis (Mot et al., 2009), the coexistence of at least one additional risk factor, such as a CVC, sepsis, asphyxia, or combined with inherited thrombophilia, leads to thrombotic complications (Berkun et al., 2014). In a review of 16 infants with perinatal thrombosis associated with maternal aPL, additional prenatal or perinatal prothrombotic factors were identified in 9 of 14 evaluable cases (64%) (Boffa and Lachassinne, 2007). These included preeclampsia, intrauterine growth restriction, asphyxia, sepsis, arterial or venous catheterization, and congenital thrombophilia. Thrombotic complications are more commonly associated with arterial thrombosis, including thrombosis of the cerebral arteries.

Elevated levels of coagulation factors, such as II, VII, VIII, IX, X, XI, fibrinogen, and VWF, are associated with an increased risk of venous thrombosis, with FVIII and VWF showing the strongest association with this risk (Rietveld et al., 2019; Koster et al., 1995; Tsai et al., 2002; Meijers et al., 2000; de Visser et al., 2001; Vlieg et al., 2000). Very high levels of FVIII and VWF are linked to a significantly increased relative risk of venous thrombosis (OR >16), while the associations of the other coagulation factors with thrombosis are mainly explained by their correlation with FVIII and VWF levels (Rietveld et al., 2019). Increased levels of these coagulation factors are not associated with an increased risk of thrombosis in neonates or children (Rietveld et al., 2019; Connors, 2017). In neonates, the haemostatic system is immature and characterized by physiologically low levels of most coagulation factors, but relatively elevated levels of FVIII and VWF, and reduced fibrinolytic activity. This contributes to the maintenance of the haemostatic balance, but the system has limited “reserves” and is more vulnerable to thrombotic stimuli, particularly in the presence of triggering factors like CVC, sepsis, or dehydration (Abdelsamei et al., 2024; Makatsariya et al., 2022; Khizroeva et al., 2023; Kenet et al., 2019a).

### 3.4 Protein C and S deficiency and neonatal purpura fulminans

Protein C deficiency (PCD) is more common during the neonatal period, whereas Protein S deficiency PSD is less frequently encountered (Hanmod et al., 2016). In its homozygous form, PCD markedly increases the risk of VTE events. In such patients VTE events typically occur immediately after birth. The clinical manifestations of these disorders primarily include thrombosis of the mesenteric veins, portal vein, renal veins, cerebral veins, and the ophthalmic vein. A characteristic feature is the development of skin necrosis, particularly shortly after the administration of vitamin K.

Homozygous PCD is extremely rare and leads to neonatal purpura fulminans. Common clinical manifestations include intrauterine brain and/or ocular injury and the appearance of skin necrosis immediately after birth. Occasionally, thrombosis of large vessels may also be present. Neonatal purpura fulminans is a rare, life-threatening condition characterized by disseminated intravascular coagulation (DIC) and haemorrhagic skin necrosis (Auletta and Headington, 1988a; Marlar et al., 1989). Cutaneous necrotic lesions may develop on the extremities and on several pressure sites such as the scalp, abdomen, buttocks, and scrotum (Auletta and Headington, 1988b; Adcock et al., 1990; Alessi et al., 1996). The condition is usually caused by homozygous or complex heterozygous deficiency of protein C and/or protein S (Alessi et al., 1996; Price et al., 2011; Sohail et al., 2024). The heterozygous parents of affected neonates typically have type I PCD but rarely present with a history of thrombotic events. Treatment includes the administration of fresh frozen plasma (FFP) 10–20 mL/kg/12 h or protein C concentrates until the resolution of the cutaneous lesions (Marlar et al., 1989; Dreyfus et al., 1991; Kroiss and Albisetti, 2010). Following initial management, protein C concentrates are considered safe and effective for prophylactic use in cases of severe congenital protein C deficiency (Srichumpuang et al., 2025; Li et al., 2025).

Protein S is an endogenous, vitamin K–dependent anticoagulant protein that functions as a cofactor for APC in the inactivation of coagulation factors Va and VIIIa (Walker, 1980). Similarly, homozygous PSD significantly increases the risk of VTE, and, as in the previous group of patients, thrombotic events typically present immediately after birth (Easterlin et al., 2022). Combined heterozygous or homozygous forms of PSD are extremely rare and usually present at birth with neonatal purpura fulminans (Pung-amritt et al., 1999; Pegelow et al., 1992). Isolated protein S concentrates are not available, so treatment consists of the administration of FFP at doses of 10–20 mL/kg/12 h) until resolution of the lesions, followed by potential long-term pharmacological anticoagulant therapy (Monagle et al., 2012).

The guideline committee of the American Society of Haematology (ASH) (Monagle et al., 2025a) has recently recommended the use of anticoagulant therapy (ACT) in combination with protein C replacement, instead of anticoagulation alone, in paediatric patients with congenital purpura fulminans due to homozygous PCD (conditional recommendation based on very low certainty of evidence). This recommendation applies to acute clinical settings, where the combination of protein C replacement and ACT is considered preferable to anticoagulant monotherapy. For long-term management, in cases where full replacement therapy with protein C is not feasible for practical or financial reasons, the combination of protein C replacement and ACT may reduce the required intensity of ACT and, consequently, lower the risk of bleeding. The role of inherited thrombophilia in TE remains poorly defined (Saracco et al., 2009). The available data in the literature are limited. However, between 20% and 33% of cases of neonatal TE are associated with the presence of inherited thrombophilia (Saracco et al., 2016a; Nowak-Göttl et al., 2011).

A systematic review of the literature showed that, although thrombophilia-predisposing conditions may contribute to the pathogenesis of neonatal TE, clinical risk factors appear to have a stronger impact on the development of the disease (Klaassen et al., 2015). This finding is particularly apparent in cases of neonatal TE associated with the presence of CVCs, where inherited thrombophilia is observed only in a small number of cases (Demirel et al., 2009; Salonvaara et al., 1999). In addition, other acquired risk factors for neonatal thrombosis include sepsis, inflammation, liver dysfunction, dehydration, prolonged hospitalization in the NICU, and the insertion of CVCs. These factors often act synergistically with inherited thrombophilias (e.g., protein C, protein S, or ATD, and FVL and PTG G20210A mutations) in the mother or the neonate, thus increasing significantly the overall risk of thrombosis.

Although certain inherited thrombophilias have been associated with an increased risk of thrombosis, the clinical utility of universal thrombophilia testing in neonates remains unclear. Particularly in clearly provoked, catheter-associated thromboses, acquired clinical factors appear to play a more important role, and thrombophilia testing is generally not expected to alter immediate therapeutic management. In contrast, a more targeted evaluation may be appropriate in cases of unprovoked or recurrent thrombosis, a positive family history, or clinical suspicion of severe inherited deficiency of natural anticoagulants (Monagle et al., 2025a; Favaloro et al., 2025; Turebylu et al., 2007). Interpretation of protein C, protein S, and antithrombin levels requires particular caution during the neonatal period because their levels are physiologically lower and age-dependent (Sokou et al., 2026a).

#### 3.4.1 Neonatal sepsis

The interaction between the inflammatory response and haemostatic mechanisms is described as an “amplification loop,” in which inflammation activates and maintains haemostatic processes, while haemostatic products in turn amplify and prolong the inflammatory response (Tsantes et al., 2023; Sokou et al., 2026b; Sokou et al., 2025c). In ill and preterm neonates, sepsis and haemostatic defects are common and may have detrimental consequences (Aarts et al., 1983; Sokou et al., 2025d). Although it is well known that these conditions often occur concurrently, the exact mechanisms regulating the interaction between inflammation and haemostasis remain largely unknown.

Sepsis increases the risk of thrombosis through activation of immune and haemostatic pathways, which share common response pathways including cellular and endothelial interactions, leading to microvascular and macrovascular thrombosis (Tsantes et al., 2023). These dysfunctions occur within the context of the developmentally distinct neonatal haemostatic system and may contribute to increased thrombotic vulnerability in preterm and critically ill neonates (Sokou et al., 2018; Sokou et al., 2022). Increased cytokine expression during the early phase of sepsis stimulates procoagulant pathways through activation of coagulation factors and consumption of anticoagulant proteins, especially *via* predominance of tissue factor (TF) expression and thrombin production. In addition, sepsis leads to suppression of the protein C pathway and increased inhibition of fibrinolytic activity (Tsantes et al., 2023).

Activation of leukocytes and platelets causes direct endothelial injury and endothelial dysfunction, promoting intraluminal stasis-disturbed blood flow, exaggerated fibrin deposition, and eventually the formation of thrombus. At the same time, components of the haemostatic system, including thrombin, factor Xa, and the TF/FVIIa complex, activate the inflammatory response through intracellular signalling pathways of endothelial cells (Iba and Levy, 2018). The result is a vicious circle where inflammation promotes coagulation, and coagulation enhances inflammation. Preterm neonates have an immature haemostatic system, characterized by low levels of natural anticoagulants (e.g., protein C, protein S, AT) and reduced fibrinolytic activity. These physiological characteristics do not, in isolation, constitute a prothrombotic state; however, their interaction with acquired clinical triggers, such as sepsis and infection, may contribute to increased thrombotic vulnerability (Sokou et al., 2023a; Sokou et al., 2018; Sokou et al., 2017).

The formation of thrombin–AT (TAT) complexes, the elevated levels of tPA, and the increased kallikrein activity observed in preterm neonates during infection reflect the simultaneous activation of the coagulation, fibrinolytic, and kinin systems. These alterations are associated with disease severity and the increased risk of developing TE complications (Brus et al., 1997; Mautone et al., 1997). The formation of TAT complexes constitutes a sensitive marker for the activation of coagulation, as thrombin is bound by AT. Elevated TAT levels indicate increased thrombin production and the establishment of a prothrombotic state (Koyama et al., 2014; Balakrishnan et al., 1991). Increased tPA levels indicate an attempt by the organism to activate fibrinolysis as a compensatory mechanism for increased coagulation (Corrigan and Jeter, 1992).

However, in preterm neonates, fibrinolytic activity is often insufficient, which leads to fibrin accumulation and an increased risk of thrombosis (Lampridou et al., 2020). Increased kallikrein activity is associated with activation of the kinin–kallikrein system, which contributes to the inflammatory response, increased vascular permeability, and further activation of coagulation, thereby deteriorating microvascular injury and thrombogenesis (Brus et al., 1997) (Figure 3).

**FIGURE 3:**
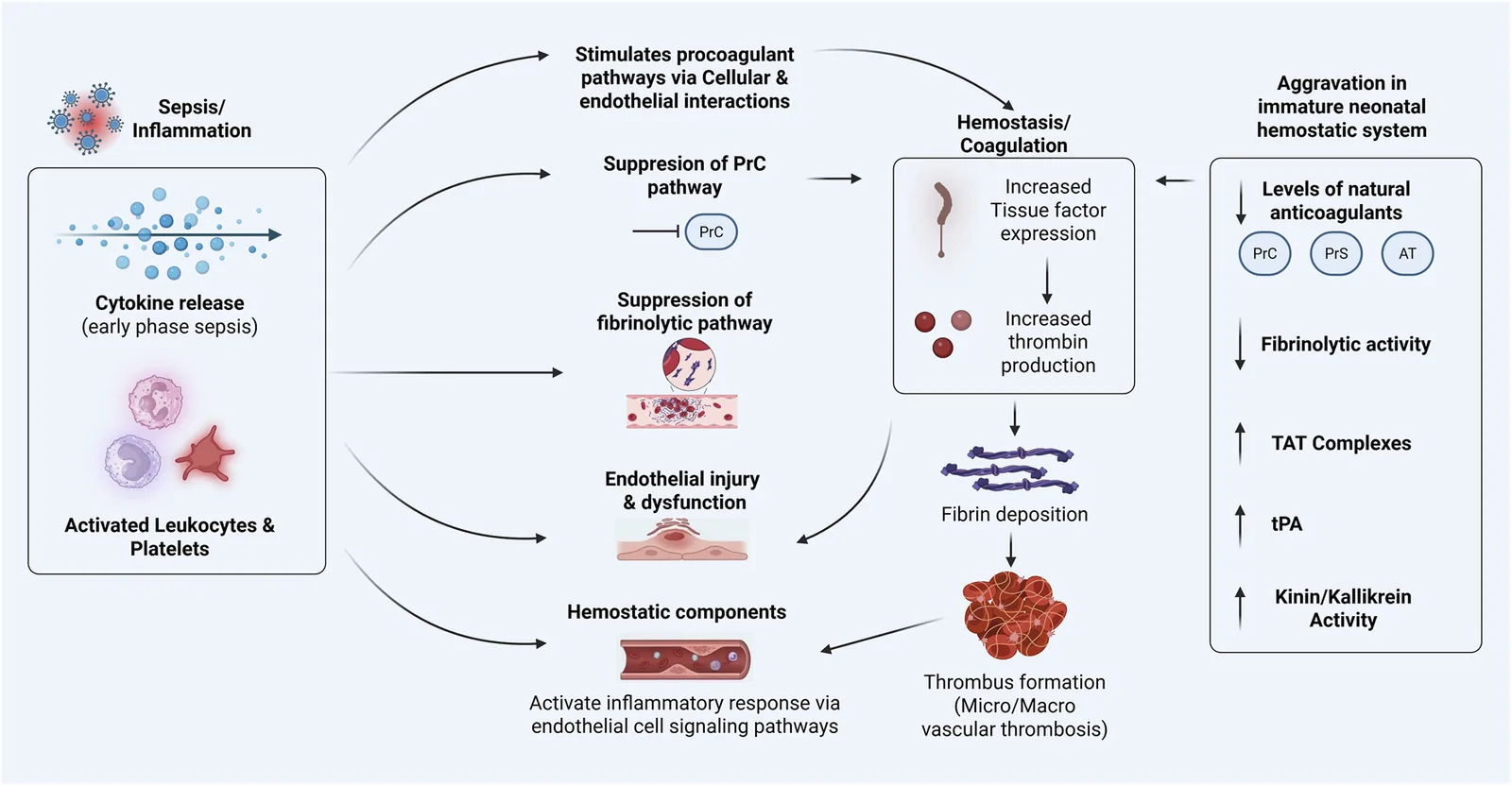
Graphical representation of the mutually amplifying loop between inflammation and coagulation in neonatal sepsis, leading to thrombus formation, aggravated by an immature hemostatic system. Abbreviations: PrC: protein C, PrS: protein S, AT: antithrombin, TAT: thrombin-antithrombin, tPA: tissue plasminogen activator. Created in BioRender. L., A. (2026) https://BioRender.com/w6ne0jv.

Sepsis-associated thrombosis presents mainly as VTE, most frequently affecting the portal vein, the superior vena cava, and the deep veins. The presence of sepsis is associated with a twofold increased risk of thrombosis in neonates, while the coexistence of additional risk factors like CVCs, prolonged hospitalization in the NICU or inherited thrombophilia has a synergistic effect, further increasing the risk (MG, 2006).

#### 3.4.2 Central vascular catheter-associated thrombosis

CVCs represent the most important acquired risk factor for thrombosis during the neonatal period (Sokou et al., 2026a; Guzman et al., 2023). The widespread use of umbilical venous catheters (UVCs) and peripherally inserted central catheters (PICCs) is essential for the care of preterm and critically ill neonates but is associated with a substantial risk of thrombosis. Approximately 85%–90% of neonatal thromboembolic events are estimated to be associated with the presence of a central catheter, although the reported incidence varies considerably based on the catheter type, the study population, and, most importantly, whether systematic imaging surveillance is performed or evaluation is undertaken only in the presence of clinical indications (Sokou et al., 2026a; Guzman et al., 2023; El-Naggar et al., 2020). Indeed, a substantial proportion of catheter-related thrombosis (CRT) is asymptomatic and is detected only on ultrasound examination, suggesting that the true incidence is likely higher than that reported in studies based exclusively on symptomatic events (Sokou et al., 2026a; Guzman et al., 2023; El-Naggar et al., 2020).

The pathogenesis of CRT is multifactorial and represents a characteristic example of the simultaneous activation of all three components of Virchow’s triad: endothelial injury, altered blood flow/stasis, and hypercoagulability (Guzman et al., 2023; Knopoff et al., 2024). Unlike other forms of neonatal thrombosis, however, the catheter itself acts as a local trigger that can simultaneously activate more than one of these mechanisms.

Endothelial injury is one of the key mechanisms initiating thrombosis. Catheter insertion causes direct mechanical trauma to the vascular endothelium, while continuous contact andmovement of the catheter tip or shaft against the vessel wall may perpetuate endothelial injury throughout the duration of catheterization (Sokou et al., 2026a). Disruption of endothelial integrity leads to exposure of subendothelial structures, platelet adhesion and activation, and local activation of the coagulation cascade, resulting in thrombin generation and fibrin deposition (Guzman et al., 2023; Knopoff et al., 2024). This injury may be exacerbated when the catheter tip is malpositioned or repeatedly comes into contact with the vascular endothelium or cardiac wall (Calandrino et al., 2025; Costa et al., 2015; Sokou et al., 2026a; Guzman et al., 2023). In addition, infusion of hyperosmolar or potentially irritant solutions through the catheter, including parenteral nutrition, certain medications, and blood products, may cause further chemical injury or endothelial activation (Guzman et al., 2023; Bhatia et al., 2022; Knopoff et al., 2024).

In parallel, the presence of the catheter substantially alters local hemodynamics. As a foreign body within the vascular lumen, the catheter disrupts normal laminar flow and creates regions of low flow velocity, turbulence, and stasis, particularly around the catheter shaft and tip (Knopoff et al., 2024). This mechanism is particularly relevant in neonates because of their small vessel diameter and the proportionally greater fraction of the vascular lumen occupied by the catheter. As the ratio of the catheter’s external diameter to the vessel diameter increases, flow obstruction and the likelihood of local stasis increase accordingly. Reduced blood flow also limits the clearance of activated coagulation factors from the catheterized region, thereby facilitating local thrombin generation and the propagation of fibrin deposition (Balasundaram, 2025; Knopoff et al., 2024).

The blood-biomaterial interaction is also of particular importance. Immediately after catheter placement, its synthetic surface comes into contact with blood and is rapidly coated with plasma proteins, including fibrinogen, fibrin, fibronectin, and other adhesive proteins. This process transforms the initially inert catheter surface into a biologically active substrate capable of facilitating platelet and cellular adhesion and further promoting coagulation activation (Knopoff et al., 2024; Latham and Thompson, 2014). Over time, a fibrin sheath may form around the catheter. The sheath may remain confined to the catheter surface or progress to a mural thrombus with extension into the adjacent vessel. Thus, CRT may manifest morphologically as pericatheter fibrin deposition, intraluminal thrombotic occlusion, or a mural/vascular thrombus, often occurring in the region of the catheter tip (Knopoff et al., 2024; Latham and Thompson, 2014).

CVCs are typically placed in neonates with coexisting prematurity, sepsis or systemic inflammation, hypoxia, dehydration, congenital heart disease, hemodynamic instability, or other serious conditions (Bhat et al., 2018; Bhat et al., 2022; El-Naggar et al., 2020; Bhatia et al., 2022). The limited reserve of the developing haemostatic system renders neonates, particularly preterm infants, more vulnerable when a strong local prothrombotic stimulus, such as a catheter, is combined with systemic activation of coagulation. Consequently, the CVC may be regarded not merely as another risk factor, but rather as a local driver of thrombogenesis in an already vulnerable host (Sokou et al., 2026a; Bhat et al., 2018; Bhat et al., 2022).

Sepsis and the presence of a CVC are two of the most important acquired risk factors for neonatal thrombosis and often act synergistically. The catheter provides a thrombogenic surface and induces local disturbances in blood flow and endothelial integrity, while catheter-related infection further enhances endothelial activation and thrombin generation, thereby increasing the risk of thrombus formation and propagation, particularly with prolonged catheterization (Gominet et al., 2017; Taylor et al., 2015; Domouchtsidou et al., 2026).

The relationship between infection and thrombosis is, in fact, bidirectional. Early deposition of fibrin and host proteins on the catheter surface creates a suitable substrate for microbial adhesion and biofilm formation (Gominet et al., 2017; Taylor et al., 2015; Domouchtsidou et al., 2026). The biofilm, together with the pre-existing thrombus or fibrin sheath, provides a protected microenvironment that facilitates microbial persistence. Conversely, the local and systemic inflammatory response to infection enhances tissue factor expression, platelet and monocyte activation, and thrombin generation, while simultaneously suppressing endogenous anticoagulant pathways and impairing fibrinolytic activity. This creates a self-reinforcing cycle of catheter-fibrin deposition-biofilm-inflammation-thrombosis, which may contribute both to persistent infection and to an increased thrombotic burden or thrombus propagation (Gominet et al., 2017; Taylor et al., 2015; Domouchtsidou et al., 2026).

The risk of CRT is also influenced by catheter-related characteristics. Prolonged catheter dwell time, malposition of the catheter tip, a larger catheter diameter relative to the vessel, the use of multilumen catheters, and the presence of multiple central lines have been associated with an increased risk of thrombosis (Sokou et al., 2026a; Viviana Bacciedoni MDa et al., 2016). The catheter-to-vessel diameter ratio appears to be particularly important, as a greater proportion of the vascular lumen occupied by the catheter results in more pronounced reduction in blood flow and local stasis (Gismondi et al., 2026; Cellini et al., 2022).

Catheter material has also been proposed as a potential modifier of thrombotic risk. Theoretically, properties such as stiffness, elasticity, surface smoothness, and chemical composition may influence both mechanical endothelial injury and protein adsorption, platelet activation, and fibrin deposition (Sokou et al., 2026a). Studies conducted predominantly in adults, including patients with haemodialysis catheters, have demonstrated differences in the biocompatibility and thrombogenic properties of various catheter materials, with silicone showing a potentially lower thrombogenic profile than polyurethane in some studies (Cellini et al., 2022; López-Rubio et al., 2024; Marin et al., 2021; Singh and Juneja, 2023).

However, the direct extrapolation of these findings to neonates is not sufficiently supported by the available evidence. Indeed, neonatal data on the impact of catheter material are extremely limited. In a recent Cochrane review (Schults et al., 2024) on PICC design and materials, the vast majority of included studies involved adults, with only one study focusing exclusively on neonates (Gilbert et al., 2019).

In the neonatal PREVAIL study (Gilbert et al., 2019), which compared antimicrobial-impregnated with conventional PICCs, clinically apparent thrombophlebitis was uncommon in both groups (4/374 [1%] vs. 7/430 [2%]). However, the study was primarily designed to evaluate infection prevention rather than thrombosis and did not include systematic imaging surveillance for asymptomatic thrombotic events. Therefore, these findings do not allow firm conclusions to be drawn regarding the effect of catheter material or antimicrobial coating on the true incidence of CRT. Similarly, the Cochrane review (Schults et al., 2024) rated the certainty of evidence as very low for the comparison between silicone and polyurethane and found no clear difference in terms of occlusion between the two materials. Consequently, the available evidence does not currently support preferential selection of a specific catheter material solely for the purpose of preventing neonatal thrombosis. At present, the use of the smallest catheter that provides adequate functionality, appropriate catheter-tip positioning, minimization of catheter dwell time, and meticulous catheter care represent more important and better-supported strategies for reducing thrombotic risk.

Overall, CRT in neonates is not merely a mechanical complication of the presence of an intravascular device. Rather, it results from a dynamic interplay among the foreign biomaterial, vascular endothelium, altered blood flow, platelet and coagulation activation, fibrin deposition, and, in some cases, microbial colonization and inflammation. This interplay explains why the risk is particularly increased when catheterization is prolonged and accompanied by infection and the overall prothrombotic burden of a critically ill neonate.

## 4 Discussion

Thrombotic complications during the neonatal period are relatively rare but increasingly recognized (Pelland-Marcotte et al., 2026). In recent years, a noteworthy rise in their incidence has been observed, which is attributed to the remarkable advances in modern neonatology, the significant achievements of intensive care, and the advanced therapeutic interventions that have dramatically improved outcomes even for the most vulnerable neonates. The survival of infants born at the limits of viability-a result of scientific and technological progress-inevitably comes with new challenges, among which is the increased frequency of thrombotic events (Song et al., 2023b). The pathophysiology and many of the risk factors associated with thromboembolic events in the neonatal period are unique to this population, highlighting the need for a stronger neonatal-specific evidence base that may inform future recommendation for prevention, diagnosis, and therapeutic management (Pelland-Marcotte et al., 2026; Song et al., 2023b).

The developmentally distinct haemostatic system of neonates differs substantially from that of adults (Sokou et al., 2023a; Sokou et al., 2025a). Despite its biological immaturity, healthy neonates preserve a delicate yet functionally adequate balance between coagulation and fibrinolysis (Sokou et al., 2023a; Suarez et al., 1984). Consequently, the neonatal haemostatic system although vulnerable, remains physiologically effective under conditions of homeostasis. However, when subjected to perinatal stressors, such as hypoxia, systemic inflammation, or sepsis, this delicate balance is easily disrupted as the limited haemostatic reserves are more rapidly exhausted, leading to an increased risk of thrombotic complications, particularly in preterm or critically ill neonates (Sokou et al., 2023a; Suarez et al., 1984).

One of the key conclusions emerging from the available evidence is that neonatal thrombosis cannot be attributed to a single pathophysiological mechanism. Rather, it appears to result from the interaction between developmental haemostasis and multiple acquired prothrombotic stimuli, including the presence of central venous catheters, sepsis and systemic inflammation, hypoxia, hemodynamic instability, and prematurity (Bhat et al., 2018; Bhat et al., 2022; El-Naggar et al., 2020; Bhatia et al., 2022).

This concept has direct clinical implications, as it shifts the approach from seeking a single underlying cause toward a comprehensive assessment of the neonate’s overall thrombotic risk. The interaction between central venous catheterization and sepsis represents a characteristic example of this multifactorial pathogenesis (Sokou et al., 2026a; Gominet et al., 2017; Taylor et al., 2015; Domouchtsidou et al., 2026). A catheter may simultaneously cause endothelial injury, disturbances in local blood flow, and activation of coagulation at its surface, while concomitant infection further enhances endothelial activation, thrombin generation, and fibrin deposition (Sokou et al., 2026a; Gominet et al., 2017; Taylor et al., 2015; Domouchtsidou et al., 2026).

The available evidence regarding the role of inherited thrombophilia further highlights the complexity of the etiological assessment of neonatal thrombosis. Although thrombophilic disorders have been reported in a proportion of neonates with thrombotic events, one or more major acquired or perinatal risk factors are present in most cases (Sokou et al., 2026a). Therefore, risk factors should not be considered as independent variables, but rather as interacting mechanisms that may act synergistically and substantially increase the overall thrombotic risk.

However, the interpretation of associations between individual risk factors and neonatal thrombosis requires caution. The available evidence is derived predominantly from retrospective or observational studies, in which factors such as prematurity, sepsis, exposure to central venous catheters, severity of the underlying illness, and duration of hospitalization frequently coexist and interact. This close interdependence limits the ability to determine the independent contribution of each factor and, most importantly, to draw firm conclusions regarding their causal relationship with the development of thrombosis.

The need for careful interpretation of individual risk factors is further supported by a recent systematic review and meta-analysis of 60 studies, which included more than 3.3 million neonates (Pelland-Marcotte et al., 2026). Preeclampsia, low birth weight, neonatal infection, and cardiac disease were significantly associated with venous thrombosis, whereas findings were inconsistent across studies for other commonly reported factors, including maternal diabetes, gestational age/prematurity, asphyxia, mechanical ventilation, and surgical procedures. Furthermore, substantial between-study heterogeneity, the lack of a standardized definition of thrombosis, and limited data for several common clinical conditions continue to hinder accurate assessment of the independent contribution of individual factors to thrombotic risk.

Once thrombosis occurs, it may further compromise the clinical condition of the neonate, contributing to prolonged hospitalization, increased morbidity and mortality, and potentially adverse long-term outcomes, especially when therapeutic intervention is not timely and evidence-based (Monagle et al., 2006; Andrew et al., 1987). Early diagnosis is a critical factor for the effective management of neonatal thrombosis (Saracco et al., 2016b; Sokou et al., 2024a; Sokou et al., 2024b; Sokou et al., 2023b; Kenet et al., 2019b). Therefore, distinguishing statistical association from demonstrated prognostic or therapeutic utility remains a critical issue that warrants further investigation before clear, neonatal-specific recommendations can be established (Sokou et al., 2026a).

A major obstacle highlighted in the current literature is the profound heterogeneity in epidemiological data and diagnostic methodologies. This heterogeneity has important implications for the interpretation of the existing literature (Pelland-Marcotte et al., 2026). Differences in reported incidence cannot be attributed solely to true differences in thrombotic risk across populations, as they are substantially influenced by the method of detection and the definition of a thrombotic event, the severity and characteristics of the study population, and whether central venous catheter-associated thromboses are included (Sokou et al., 2026a).

Systematic imaging surveillance identifies more-usually asymptomatic-thrombotic events, compared with clinically guided evaluation, whereas studies based on administrative or clinical databases may underestimate or misclassify thrombotic events. Consequently, the true burden of neonatal thrombosis remains uncertain. At the same time, the clinical significance and natural history of some asymptomatic thrombotic events have not yet been fully elucidated. The development of standardized definitions, diagnostic criteria, and comparable imaging protocols is therefore essential for generating reliable epidemiological data.

From a therapeutic perspective, the heterogeneity of neonatal thrombosis and the limited availability of neonatal-specific evidence necessitate an individualized approach to management (Sokou et al., 2026a; Monagle et al., 2025b). The decision to initiate anticoagulant therapy depends on the location and extent of the thrombus, the presence of symptoms or thrombus progression, underlying risk factors, and bleeding risk. LMWH is the most established anticoagulant option in neonates; however, age-dependent pharmacokinetic and pharmacodynamic characteristics during the neonatal period may result in different dosing requirements compared with older children, making dose individualization and monitoring of anti-Xa activity necessary (Ferriero et al., 2019; Monagle et al., 2025b; Samji et al., 2022; Grizante-Lopes et al., 2020; Raffini, 2009; Woods et al., 2021). Concurrently, bleeding risk should be carefully evaluated, taking into account gestational age, platelet count, pre-existing intracranial haemorrhage, hepatic function, and other clinical factors that may affect the safety of anticoagulant therapy. Ongoing management should include serial imaging to monitor thrombus progression or resolution, together with anti-factor Xa monitoring, when indicated, to guide anticoagulant dosing and ensure therapeutic levels (Ferriero et al., 2019; Monagle et al., 2025b; Samji et al., 2022; Grizante-Lopes et al., 2020; Raffini, 2009; Woods et al., 2021). UFH may be used in selected clinical circumstances. Treatment duration should be individualized, whereas thrombolysis is generally reserved for severe, life-threatening, limb-threatening, or organ-threatening thromboses, given the increased risk of bleeding and the limited neonatal evidence (Sokou et al., 2026a; Ferriero et al., 2019; Monagle et al., 2025b; Samji et al., 2022; Grizante-Lopes et al., 2020; Raffini, 2009; Woods et al., 2021).

In parallel, newer anticoagulant strategies, including direct thrombin inhibitors and direct oral anticoagulants, have expanded the therapeutic options available for pediatric thrombosis. However, experience with these agents during the neonatal period remains extremely limited, particularly in preterm infants, and specifically designed pharmacokinetic and clinical studies are needed before their broader use can be considered in this population (Khizroeva et al., 2023; Sokou et al., 2024b; FDA, 2025; Jaffray and Young, 2022; Anthony et al., 2025). The recent International Society on Thrombosis and Haemostasis Scientific and Standardization Committee (ISTH SSC) guidance on the development of antithrombotic drugs for the pediatric population further emphasizes the need for age-appropriate pharmacokinetic and pharmacodynamic evaluation, suitable pediatric/neonatal formulations, and adequate pediatric-specific evidence, particularly given the developmental differences in hemostasis that may influence drug response (Goldenberg et al., 2026).

A recent international survey conducted by the ISTH SSC demonstrated substantial heterogeneity in the management of catheter-associated venous thromboembolism in neonates, both with regard to the decision to initiate anticoagulant therapy and its duration (Bhat et al., 2026). The variability in clinical practice seems to extend beyond acute management. In a separate recent international ISTH SSC survey (ASTRO-Kids), substantial variation was observed in long-term follow-up practices after VTE, with particularly limited follow-up in the neonatal population (Cohen et al., 2026). The small number of cases managed annually at most centers represents an additional major barrier to the accumulation of sufficient data and the development of robust evidence specifically for the neonatal population (Bhat et al., 2026). Overall, the findings of these two studies highlight gaps at different stages of care and support the need for more standardized approaches, prospective studies, and international multicenter collaboration (Bhat et al., 2026; Cohen et al., 2026).

In this context, a thorough understanding of the epidemiology and distinctive pathophysiological mechanisms of neonatal thrombosis represents an essential first step toward the development of evidence-based, neonatal-specific recommendations in the future. However, translating this knowledge into clinical recommendations is currently constrained by the heterogeneity of the available studies, the lack of standardized definitions and diagnostic approaches, and the relative paucity of prospective, multicenter studies specifically designed for the neonatal population. These gaps are consistent with the recently identified research priorities of the ISTH SSC for pediatric VTE, which emphasize improving the identification and stratification of risk factors, developing and prospectively validating risk assessment models, and optimizing thromboprophylaxis strategies, while also recognizing the continued underrepresentation of neonates in clinical studies (Whitworth et al., 2026). Addressing these gaps is an essential prerequisite for the future development of evidence-based guidelines that reflect the unique biological and clinical needs of neonates, rather than relying primarily on extrapolation of data from older children and adults.

## 5 Conclusions and future directions

Neonatal thromboembolic disease is a complex and increasingly recognized clinical entity. The available evidence supports a multifactorial pathogenesis in which the distinctive features of developmental haemostasis interact with multiple acquired, maternal, placental, and perinatal risk factors. Despite the clinical significance of thrombotic events and their association with increased morbidity and mortality, the true epidemiological burden of neonatal thrombosis remains incompletely characterized.

More accurate characterization of the epidemiology and a deeper understanding of the distinctive pathophysiological mechanisms underlying neonatal thrombosis represent fundamental steps toward establishing a stronger evidence base specific to the neonatal population. Addressing existing methodological and knowledge gaps could strengthen the evidence supporting future strategies for prevention, diagnosis, and management and, ultimately, contribute to the development of evidence-based guidelines tailored to the unique biological and clinical needs of neonates, thereby reducing reliance on data derived from older children and adults.

Finally, systematic documentation and long-term follow-up of neonates after a thrombotic event could contribute to a more comprehensive understanding of the natural history of the disease and to a more accurate assessment of the risk of recurrence, late vascular complications, and long-term neurodevelopmental outcomes.
